# Supplementary figures and images for: Transcriptomic study of the mechanism of anoikis resistance in head and neck squamous carcinoma
Source: PeerJ. 2019 May 23;7:e6978. doi: 10.7717/peerj.6978 (PMC6535219; doi:10.7717/peerj.6978)

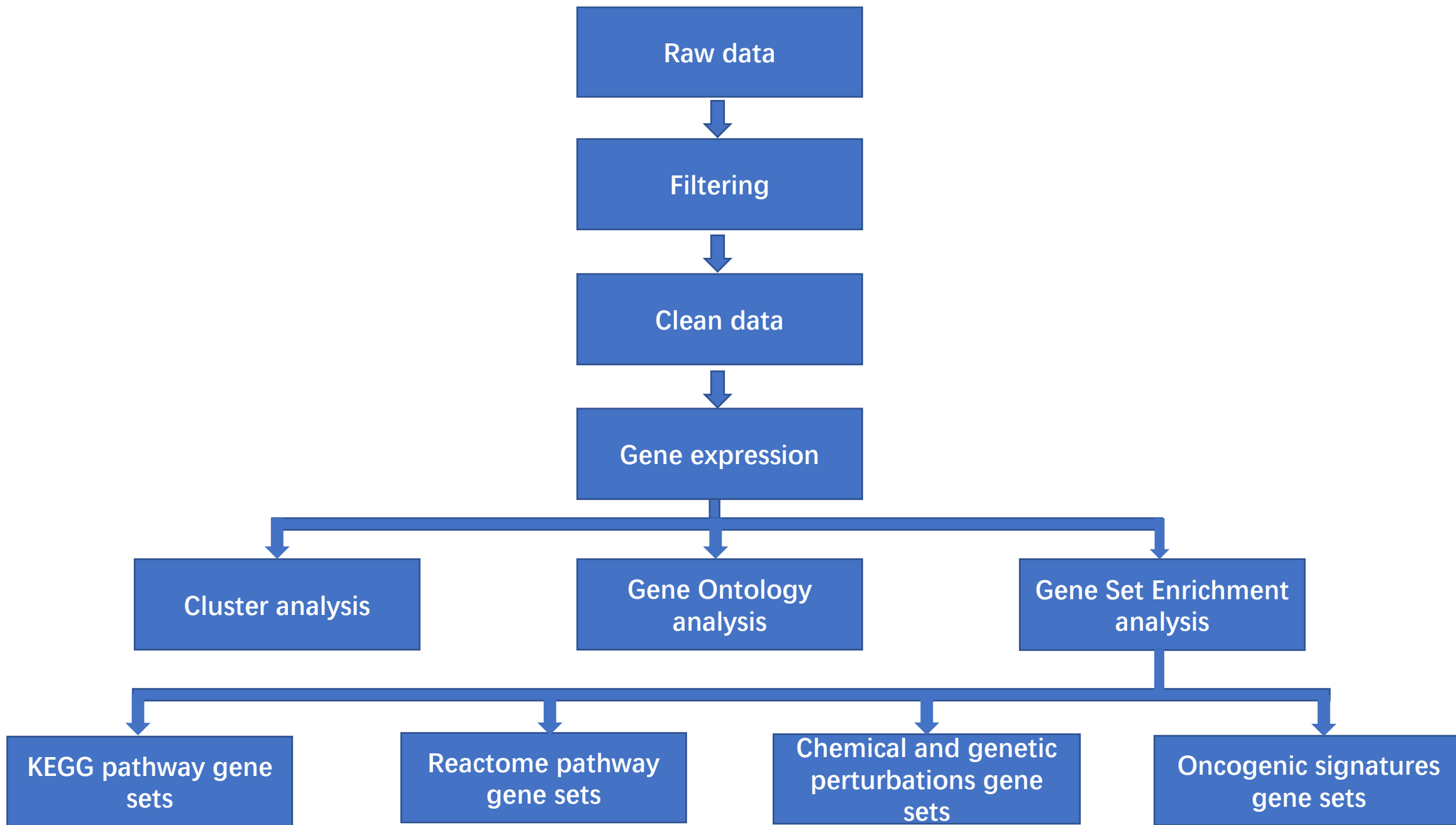

Supplement: Figure S1 [file peerj-07-6978-s002.pdf]

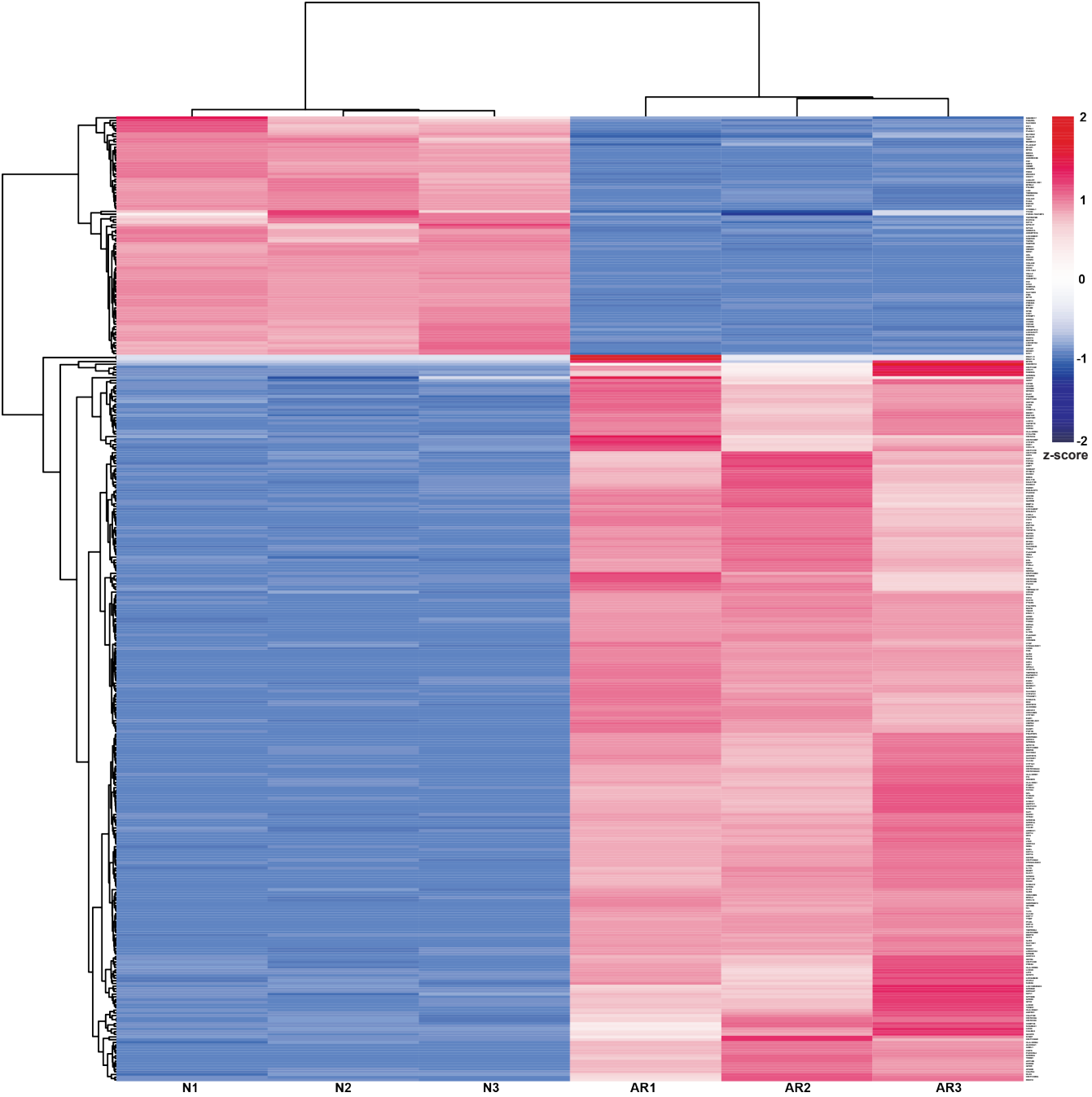

Supplement: Figure S2 — Cluster heatmap of DEGs (|Loget| ≥ 3, probability ≥ 0.9) was generated by OmicShare tools (http://www.omicshare.com/tools/). The color of the squares in the heatmap reflects the z-score. Cluster of genes showing representative expression patterns between normal CAL27 cells and anoikis-resistant CAL27 cells. [file peerj-07-6978-s003.pdf]

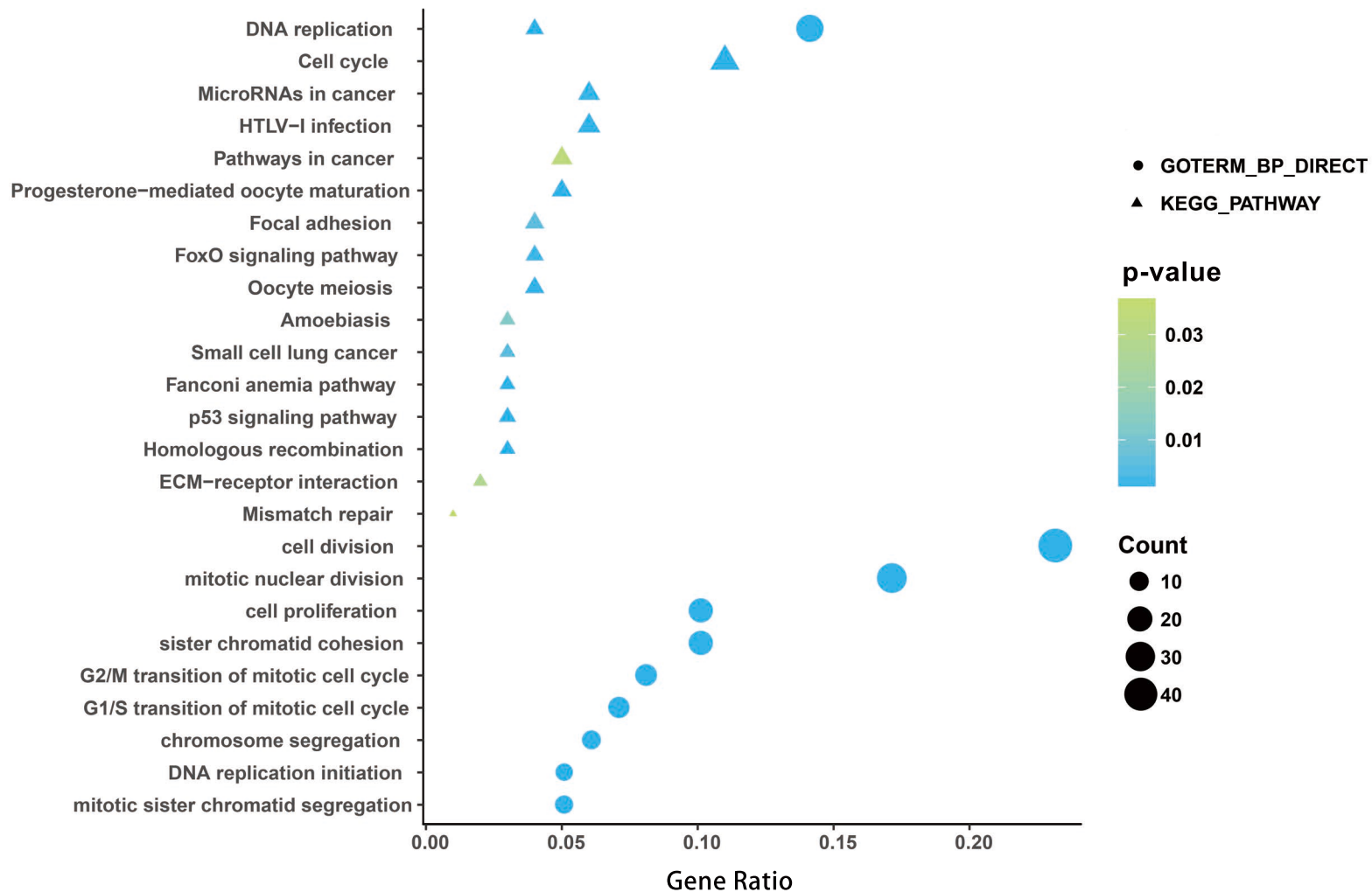

Supplement: Figure S3 — Most significantly down-regulated genes were associated with protein binding, however their biological function was unclear. So we further re-analyzed these genes related to protein binding using DAVID. The results of gene biological process and KEGG enrichment analysis for DEGs were related to protein binding molecular function.The results revealed that most down-regulated genes related to protein binding influenced cell cycle ,DNA replication and the p53 signaling pathway. p-value ≤ 0.05 and FDR ≤ 0.05. [file peerj-07-6978-s004.pdf]
