# Supplementary material for: Transcriptomic study of the mechanism of anoikis resistance in head and neck squamous carcinoma
Source: PeerJ. 2019 May 23;7:e6978. doi: 10.7717/peerj.6978 (PMC6535219; doi:10.7717/peerj.6978)
Supplement: Table S1 [file peerj-07-6978-s005.docx]

| Gene | Forward primer | Reverse primer | Annealing temperature ( ℃ ) | Amplification cycles |
| --- | --- | --- | --- | --- |
| KRT9 | GGGGCCGATTCAGCTCTTC | CTACTGGCACTAAAACCACCC | 51 | 40 |
| KRT80 | CCTCCCTAATTGGCAAGGTG | AGATGCCCGAGGTCGAAGAT | 54 | 40 |
| KRT8 | CAGAAGTCCTACAAGGTGTCCA | CTCTGGTTGACCGTAACTGCG | 51 | 40 |
| KRTDAP | CCATTGAGAATTATGCGTCACGA | AAGAGGGCGTGCCAGTTCAG | 56 | 40 |
| KRT16 | GACCGGCGGAGATGTGAAC | CTGCTCGTACTGGTCACGC | 52 | 40 |
| KRT17 | GGTGGGTGGTGAGATCAATGT | CGCGGTTCAGTTCCTCTGTC | 54 | 40 |
| KRT18 | GGCATCCAGAACGAGAAGGAG | ATTGTCCACAGTATTTGCGAAGA | 54 | 40 |
| KRT5 | CCAAGGTTGATGCACTGATGG | TGTCAGAGACATGCGTCTGC | 52 | 40 |
| CDH11 | AGAGAGCCCAGTACACGTTGA | TTGGCATGATAGGTCTCGTGC | 60 | 40 |
| KRT14 | TGAGCCGCATTCTGAACGAG | GATGACTGCGATCCAGAGGA | 52 | 40 |
| KRT15 | TCTGCTAGGTTTGTCTCTTCAGG | CCAGGGCACGTACCTTGTC | 52 | 40 |
| S100A12 | AGCATCTGGAGGGAATTGTCA | GCAATGGCTACCAGGGATATGAA | 58 | 40 |
| SPRR1A | CCCTTCAACGGTCACTCCAG | AGAGGTGCAAAGGAGCGATT | 55 | 40 |
| SPRR2E | CCAGAGCTTTGGAAGAAGGAC | AAAGTGACAATTGCACAGGTGTT | 54 | 40 |
| RPTN | ATGGGGACTGTGCCTTACTAT | TCTCGGTCTTGATCTAAGAGGTT | 52 | 40 |
| LRRN4 | ACGCTCGAAGTCCTGGATCT | TCCTCAGGTAGAGGGATGTGA | 54 | 40 |
| ADAMTS1 | ACTGGAAGCATAAGAAAGAAGCG | AATTCTGCCATCGACTGGTCT | 54 | 40 |
| ALOX5AP | TCAGCGTGGTCCAGAATGG | GCAAGTGTTCCGGTCCTCT | 54 | 40 |
| KRT10 | ATGTCTGTTCGATACAGCTCAAG | CTCCACCAAGGGAGCCTTTG | 56 | 40 |
| SPRR4 | GGCTCACCTGTTCCTAGAGC | CTTCTGCTGGGCTGGAATGA | 56 | 40 |
| THBS1 | AGACTCCGCATCGCAAAGG | TCACCACGTTGTTGTCAAGGG | 56 | 40 |
| KLF4 | CAGCTTCACCTATCCGATCCG | GACTCCCTGCCATAGAGGAGG | 62 | 38 |
| IL6 | ACTCACCTCTTCAGAACGAATTG | CCATCTTTGGAAGGTTCAGGTTG | 62 | 35 |
| VEGFA | AGGGCAGAATCATCACGAAGT | AGGGTCTCGATTGGATGGCA | 60 | 35 |
| PDGFA | GCAAGACCAGGACGGTCATTT | GGCACTTGACACTGCTCGT | 58 | 35 |
| CDKN1A | TGTCCGTCAGAACCCATGC | AAAGTCGAAGTTCCATCGCTC | 60 | 35 |
| TP53 | CAGCACATGACGGAGGTTGT | TCATCCAAATACTCCACACGC | 60 | 35 |
| EGFR | TTGCCGCAAAGTGTGTAACG | GTCACCCCTAAATGCCACCG | 62 | 36 |
| GAPDH | GAAGGTGAAGGTCGGAGTC | GAAGATGGTGATGGGATTTC | 50 | 28 |

**Table of primers for RT-PCR**
