# Supplementary material for: Transcriptomic study of the mechanism of anoikis resistance in head and neck squamous carcinoma
Source: PeerJ. 2019 May 23;7:e6978. doi: 10.7717/peerj.6978 (PMC6535219; doi:10.7717/peerj.6978)
Supplement: Table S2 [file peerj-07-6978-s006.docx]

**Up-regulated genes related to the “extracellular exosome” term.**

| **Symbol** | **Description** | **Gene Length** | **Loget** | **Probability** |
| --- | --- | --- | --- | --- |
| KRT1 | keratin 1 | 5672 | 12.30349538 | 0.99923305 |
| KRT13 | keratin 13 | 4725 | 10.47762855 | 0.99987658 |
| SPRR3 | small proline-rich protein 3 | 2110 | 10.24965219 | 0.99687048 |
| KRT14 | keratin 14 | 4643 | 9.953820604 | 0.99991184 |
| SPRR1B | small proline-rich protein 1B | 1699 | 9.884305631 | 0.99996474 |
| RHCG | Rh family, C glycoprotein | 25216 | 9.660673191 | 0.99987658 |
| MTPN | myotrophin | 50702 | 8.938599455 | 0.95928982 |
| KLK12 | kallikrein-related peptidase 12 | 6693 | 8.825488851 | 0.95526112 |
| IVL | involucrin | 3342 | 8.732119776 | 0.99891569 |
| S100A7 | S100 calcium binding protein A7 | 2958 | 8.708591404 | 0.99993829 |
| PI3 | peptidase inhibitor 3, skin-derived | 1669 | 8.476759455 | 0.99989421 |
| KRT9 | keratin 9 | 6218 | 8.20701432 | 0.967286 |
| S100A8 | S100 calcium binding protein A8 | 32547 | 8.192729616 | 0.9998854 |
| ARSD | arylsulfatase D | 25406 | 8.118941073 | 0.94213477 |
| LYPD2 | LY6/PLAUR domain containing 2 | 2385 | 7.994353437 | 0.9099933 |
| CALML5 | calmodulin-like 5 | 876 | 7.701595261 | 0.97853415 |
| A2ML1 | alpha-2-macroglobulin-like 1 | 64530 | 7.6794801 | 0.94879051 |
| LYNX1 | Ly6/neurotoxin 1 | 13515 | 7.559695742 | 0.99430516 |
| CLCA4 | chloride channel accessory 4 | 33683 | 7.494188905 | 0.988875 |
| KRT16 | keratin 16 | 6122 | 7.31065424 | 0.99943581 |
| S100P | S100 calcium binding protein P | 4102 | 7.10433666 | 0.98051765 |
| BBOX1 | butyrobetaine (gamma), 2-oxoglutarate dioxygenase (gamma-butyrobetaine hydroxylase) 1 | 87105 | 6.925352131 | 0.952969 |
| FXYD2 | FXYD domain containing ion transport regulator 2 | 27855 | 6.87036472 | 0.97085581 |
| CEACAM5 | carcinoembryonic antigen-related cell adhesion molecule 5 | 21849 | 6.722400966 | 0.98857506 |
| KLK11 | kallikrein-related peptidase 11 | 5824 | 6.710713372 | 0.993521 |
| KRT15 | keratin 15 | 8787 | 6.553334845 | 0.99861596 |
| HIST2H2BF | histone cluster 2, H2bf | 85020 | 6.438197784 | 0.976128 |
| S100A9 | S100 calcium binding protein A9 | 3174 | 6.330212345 | 0.998202 |
| SBSN | suprabasin | 4985 | 6.017637661 | 0.98091435 |
| IL1RN | interleukin 1 receptor antagonist | 34652 | 5.892748394 | 0.99507211 |
| HIST1H4H | histone cluster 1, H4h | 8030 | 5.850960567 | 0.98987976 |
| HIST1H1E | histone cluster 1, H1e | 785 | 5.828990836 | 0.902676 |
| SPINK5 | serine peptidase inhibitor, Kazal type 5 | 111684 | 5.77489871 | 0.99054092 |
| AKR1C1 | aldo-keto reductase family 1, member C1 | 20031 | 5.622496487 | 0.9963151 |
| HIST2H2AA3 | histone cluster 2, H2aa3 | 564 | 5.608548699 | 0.99527487 |
| HIST2H2AA4 | histone cluster 2, H2aa4 | 564 | 5.608548699 | 0.99527487 |
| KRT6C | keratin 6C | 5270 | 5.572251449 | 0.96433231 |
| HIST1H2BG | histone cluster 1, H2bg | 1534 | 5.540335168 | 0.99032935 |
| HIST2H2BE | histone cluster 2, H2be | 2224 | 5.380977143 | 0.994261 |
| GCNT3 | glucosaminyl (N-acetyl) transferase 3, mucin type | 45365 | 5.227168953 | 0.98323284 |
| HIST1H2BC | histone cluster 1, H2bc | 9054 | 5.204403387 | 0.992533 |
| PLXDC2 | plexin domain containing 2 | 473630 | 5.189824559 | 0.908283 |
| PKP1 | plakophilin 1 (ectodermal dysplasia/skin fragility syndrome) | 49542 | 4.934848157 | 0.993468 |
| PRSS3 | protease, serine, 3 | 48766 | 4.82920379 | 0.986812 |
| LOXL4 | lysyl oxidase-like 4 | 20565 | 4.773609668 | 0.99013541 |
| UGT1A6 | UDP glucuronosyltransferase 1 family, polypeptide A6 | 81699 | 4.755770424 | 0.98696181 |
| CKMT1A | creatine kinase, mitochondrial 1A | 6337 | 4.750427854 | 0.98584224 |
| HIST1H3E | histone cluster 1, H3e | 3275 | 4.739634781 | 0.96706513 |
| KRT6B | keratin 6B | 5476 | 4.672013177 | 0.99231285 |
| CKMT1B | creatine kinase, mitochondrial 1B | 12040 | 4.659975855 | 0.96784971 |
| HIST1H4E | histone cluster 1, H4e | 1487 | 4.649502753 | 0.94236398 |
| WNT4 | wingless-type MMTV integration site family, member 4 | 26677 | 4.590330968 | 0.98211326 |
| KRT6A | keratin 6A | 6224 | 4.575321623 | 0.992489 |
| KRT17 | keratin 17 | 5406 | 4.476743195 | 0.991775 |
| NEBL | nebulette | 394217 | 4.448685237 | 0.92805635 |
| KRT10 | keratin 10 | 4502 | 4.447140743 | 0.99102578 |
| HIST1H2BD | histone cluster 1, H2bd | 13507 | 4.416228949 | 0.99021475 |
| FXYD3 | FXYD domain containing ion transport regulator 3 | 8509 | 4.382738971 | 0.99076131 |
| SDCBP2 | syndecan binding protein (syntenin) 2 | 19331 | 4.361026141 | 0.98589513 |
| HTRA1 | HtrA serine peptidase 1 | 53384 | 4.325797907 | 0.98094961 |
| SLPI | secretory leukocyte peptidase inhibitor | 2326 | 4.317619281 | 0.99076131 |
| CNFN | cornifelin | 3299 | 4.307955152 | 0.985701 |
| CDSN | corneodesmosin | 5388 | 4.123679505 | 0.97336824 |
| GGT6 | gamma-glutamyltransferase 6 | 3892 | 4.107506922 | 0.97176381 |
| HIST4H4 | histone cluster 4, H4 | 3133 | 4.105375477 | 0.95749145 |
| SERPINB13 | serpin peptidase inhibitor, clade B (ovalbumin), member 13 | 17651 | 4.067662599 | 0.98461688 |
| HIST1H3D | histone cluster 1, H3d | 2510 | 4.023742089 | 0.95430022 |
| HIST1H2AC | histone cluster 1, H2ac | 14972 | 3.885890722 | 0.98408794 |
| CRYAB | crystallin, alpha B | 15158 | 3.882643049 | 0.90986107 |
| DUOX2 | dual oxidase 2 | 21695 | 3.863378289 | 0.93593744 |
| TMC4 | transmembrane channel-like 4 | 13156 | 3.823662235 | 0.98190169 |
| AKR1B10 | aldo-keto reductase family 1, member B10 (aldose reductase) | 13823 | 3.735522177 | 0.965734 |
| PGAM2 | phosphoglycerate mutase 2 (muscle) | 3246 | 3.734340125 | 0.957218 |
| GPX2 | glutathione peroxidase 2 (gastrointestinal) | 3754 | 3.612471372 | 0.976894 |
| ANO1 | anoctamin 1, calcium activated chloride channel | 111244 | 3.605530803 | 0.9693748 |
| SULT2B1 | sulfotransferase family, cytosolic, 2B, member 1 | 47535 | 3.599014184 | 0.967779 |
| CALB1 | calbindin 1, 28kDa | 36868 | 3.553505069 | 0.90872386 |
| CD14 | CD14 molecule | 1974 | 3.520047472 | 0.97026517 |
| PIK3IP1 | phosphoinositide-3-kinase interacting protein 1 | 10942 | 3.469681715 | 0.954089 |
| MYH14 | myosin, heavy chain 14, non-muscle | 122359 | 3.460373172 | 0.977203 |
| HIST2H4A | histone cluster 2, H4a | 7111 | 3.459431619 | 0.97156987 |
| HIST2H4B | histone cluster 2, H4b | 7166 | 3.459431619 | 0.97156987 |
| HIST1H2AE | histone cluster 1, H2ae | 564 | 3.45532722 | 0.9323407 |
| AKR1C3 | aldo-keto reductase family 1, member C3 | 72333 | 3.443503141 | 0.9763479 |
| HIST2H3C | histone cluster 2, H3c | 1656 | 3.332000903 | 0.93640467 |
| HIST2H3A | histone cluster 2, H3a | 1656 | 3.332000903 | 0.936405 |
| S100A4 | S100 calcium binding protein A4 | 6524 | 3.308299455 | 0.97340351 |
| TNFSF10 | tumor necrosis factor (ligand) superfamily, member 10 | 18000 | 3.231147111 | 0.97495504 |
| HLA-DRB5 | major histocompatibility complex, class II, DR beta 5 | 12974 | 3.195483412 | 0.96680948 |
| SCEL | sciellin | 109590 | 3.158520238 | 0.96619239 |
| HLA-DRB1 | major histocompatibility complex, class II, DR beta 1 | 11080 | 3.091686182 | 0.97008005 |
| CTSF | cathepsin F | 5379 | 3.01832251 | 0.96596319 |
| SERPINB3 | serpin peptidase inhibitor, clade B (ovalbumin), member 3 | 6767 | 3.009125733 | 0.9704503 |
| ERV3-1 | endogenous retrovirus group 3, member 1 | 16392 | 3.003493102 | 0.96907507 |
| CLIC3 | chloride intracellular channel 3 | 2196 | 2.974451204 | 0.952687 |
| BTG2 | BTG family, member 2 | 4112 | 2.973301027 | 0.96299235 |
| HLA-DRA | major histocompatibility complex, class II, DR alpha | 5205 | 2.96559011 | 0.967735 |
| LCN2 | lipocalin 2 | 4385 | 2.948880338 | 0.968097 |
| BST2 | bone marrow stromal cell antigen 2 | 2710 | 2.927394943 | 0.96788497 |
| SYT7 | synaptotagmin VII | 74681 | 2.926950076 | 0.95376247 |
| TGM1 | transglutaminase 1 | 15319 | 2.870802801 | 0.91805952 |
| GLUL | glutamate-ammonia ligase | 14114 | 2.850062668 | 0.96769103 |
| SERPINB4 | serpin peptidase inhibitor, clade B (ovalbumin), member 4 | 7061 | 2.842973182 | 0.96532847 |
| PLA2R1 | phospholipase A2 receptor 1, 180kDa | 130901 | 2.824512043 | 0.917522 |
| VAV3 | vav 3 guanine nucleotide exchange factor | 393985 | 2.789460801 | 0.950607 |
| HIST1H2AI | histone cluster 1, H2ai | 503 | 2.761918967 | 0.918253 |
| SERINC5 | serine incorporator 5 | 144856 | 2.752102583 | 0.95798512 |
| TMPRSS2 | transmembrane protease, serine 2 | 66566 | 2.707536117 | 0.92927289 |
| S100A14 | S100 calcium binding protein A14 | 2732 | 2.696146604 | 0.962393 |
| ACP5 | acid phosphatase 5, tartrate resistant | 4349 | 2.674795002 | 0.95930745 |
| SERPING1 | serpin peptidase inhibitor, clade G (C1 inhibitor), member 1 | 17476 | 2.659142754 | 0.95050954 |
| CSTA | cystatin A (stefin A) | 16809 | 2.630774741 | 0.96238408 |
| FUT3 | fucosyltransferase 3 (galactoside 3(4)-L-fucosyltransferase, Lewis blood group) | 8587 | 2.627796933 | 0.956848 |
| DSG3 | desmoglein 3 | 30934 | 2.622222697 | 0.959061 |
| HIST1H3H | histone cluster 1, H3h | 1237 | 2.588888204 | 0.93153 |
| CFB | complement factor B | 6435 | 2.550904411 | 0.95590465 |
| CTSC | cathepsin C | 44196 | 2.529075966 | 0.955861 |
| ITM2B | integral membrane protein 2B | 37220 | 2.525905211 | 0.95588702 |
| RAB15 | RAB15, member RAS oncogene family | 27413 | 2.523982874 | 0.91347544 |
| S100A6 | S100 calcium binding protein A6 | 1646 | 2.523134743 | 0.95592228 |
| MUC16 | mucin 16, cell surface associated | 132499 | 2.515203584 | 0.91082196 |
| CST3 | cystatin C | 11768 | 2.509762624 | 0.955799 |
| ANXA9 | annexin A9 | 14047 | 2.424922088 | 0.92240559 |
| FUCA1 | fucosidase, alpha-L- 1, tissue | 23293 | 2.414653753 | 0.94814697 |
| PSCA | prostate stem cell antigen | 12420 | 2.413504697 | 0.9230932 |
| CTSD | cathepsin D | 11822 | 2.393500261 | 0.95512 |
| LGALS3 | lectin, galactoside-binding, soluble, 3 | 21321 | 2.365422136 | 0.95448535 |
| LGALS9 | lectin, galactoside-binding, soluble, 9 | 19763 | 2.304378623 | 0.93126521 |
| SERPINB1 | serpin peptidase inhibitor, clade B (ovalbumin), member 1 | 9718 | 2.270789752 | 0.94648965 |
| ATP1B3 | ATPase, Na+/K+ transporting, beta 3 polypeptide | 50417 | 2.252937624 | 0.94700095 |
| ARHGAP23 | Rho GTPase activating protein 23 | 93113 | 2.219019551 | 0.941394 |
| TIMP3 | TIMP metallopeptidase inhibitor 3 | 62228 | 2.203758597 | 0.93094785 |
| CPE | carboxypeptidase E | 137137 | 2.185244273 | 0.926681 |
| NDRG2 | NDRG family member 2 | 54110 | 2.180630794 | 0.946199 |
| FAT2 | FAT tumor suppressor homolog 2 (Drosophila) | 148049 | 2.146986587 | 0.94386262 |
| GSN | gelsolin | 125051 | 2.105447296 | 0.94396841 |
| SLC9A3R1 | solute carrier family 9, subfamily A (NHE3, cation proton antiporter 3), member 3 regulator 1 | 20749 | 2.100779931 | 0.943536 |
| SLC37A2 | solute carrier family 37 (glycerol-3-phosphate transporter), member 2 | 27450 | 2.091652872 | 0.930005 |
| HSPA2 | heat shock 70kDa protein 2 | 10269 | 2.090648282 | 0.94184386 |
| METRNL | meteorin, glial cell differentiation regulator-like | 15439 | 2.080465711 | 0.93238478 |
| SGSH | N-sulfoglucosamine sulfohydrolase | 14208 | 2.070149099 | 0.914022 |
| AKR1B1 | aldo-keto reductase family 1, member B1 (aldose reductase) | 16935 | 2.068883866 | 0.94509679 |
| HSPB1 | heat shock 27kDa protein 1 | 1754 | 2.053765445 | 0.94484114 |
| DSP | desmoplakin | 45143 | 2.033800108 | 0.94381854 |
| TMEM106B | transmembrane protein 106B | 32146 | 2.02480701 | 0.94121796 |
| DSC2 | desmocollin 2 | 43593 | 2.020606301 | 0.93258754 |
