# Supplementary material for: Transcriptomic study of the mechanism of anoikis resistance in head and neck squamous carcinoma
Source: PeerJ. 2019 May 23;7:e6978. doi: 10.7717/peerj.6978 (PMC6535219; doi:10.7717/peerj.6978)
Supplement: Table S3 — Table A shows the activated REACTOME pathways in CAL27AR cells. Table B shows the repressive REACTOME pathways in CAL27AR cells. [file peerj-07-6978-s007.docx]

**Results of Reactome pathways analysis based on GSEA.**

**A**

| **PATHWAY** | **SIZE** | **ES** | **NOM p-val** | **FDR q-val** |
| --- | --- | --- | --- | --- |
| REACTOME_RNA_POL_I_PROMOTER_  OPENING | 39 | 0.7571808 | 0 | 0 |
| REACTOME_INTERFERON_  ALPHA_BETA_SIGNALING | 47 | 0.7390151 | 0 | 0 |
| REACTOME_PACKAGING_OF_  TELOMERE_ENDS | 30 | 0.7253221 | 0 | 0 |
| REACTOME_METABOLISM_OF_STEROID_  HORMONES_AND_VITAMINS_A_AND_D | 16 | 0.72153693 | 0.001831502 | 0.002075139 |
| REACTOME_GENERATION_OF_  SECOND_MESSENGER_MOLECULES | 19 | 0.71909744 | 0 | 0.001035765 |
| REACTOME_AMYLOIDS | 49 | 0.69897753 | 0 | 0 |
| REACTOME_CYTOCHROME_  P450_ARRANGED_BY_SUBSTRATE_TYPE | 24 | 0.679377 | 0 | 8.54E-04 |
| REACTOME_AMINE_COMPOUND_  SLC_TRANSPORTERS | 16 | 0.6727143 | 0.001872659 | 0.009288154 |
| REACTOME_INTERFERON_  GAMMA_SIGNALING | 52 | 0.6550511 | 0 | 0 |
| REACTOME_PHASE1_FUNCTIONALIZATION_  OF_COMPOUNDS | 34 | 0.64593416 | 0 | 1.08E-04 |
| REACTOME_MEIOTIC_SYNAPSIS | 50 | 0.60007626 | 0 | 3.19E-04 |
| REACTOME_DOWNSTREAM_TCR_SIGNALING | 30 | 0.582416 | 0 | 0.008053294 |
| REACTOME_RNA_POL_I_TRANSCRIPTION | 64 | 0.56907827 | 0 | 4.33E-04 |
| REACTOME_BIOLOGICAL_OXIDATIONS | 74 | 0.5608006 | 0 | 3.64E-04 |
| REACTOME_MEIOTIC_RECOMBINATION | 59 | 0.5248495 | 0 | 0.004610789 |
| REACTOME_INTERFERON_SIGNALING | 133 | 0.48589844 | 0 | 9.57E-04 |
| REACTOME_MEIOSIS | 84 | 0.47009262 | 0 | 0.009622734 |

**Activated REACTOME pathways**

**B**

| **PATHWAY** | | **SIZE** | **ES** | **NOM p-val** | **FDR q-val** |
| --- | --- | --- | --- | --- | --- |
| REACTOME_G1_S_SPECIFIC_  TRANSCRIPTION | 16 | -0.8642605 | 0 | 0 |  |
| REACTOME_DNA_STRAND_  ELONGATION | | 30 | -0.8639629 | 0 | 0 |
| REACTOME_EXTENSION_OF_  TELOMERES | | 27 | -0.8303774 | 0 | 0 |
| REACTOME_LAGGING_STRAND_  SYNTHESIS | | 19 | -0.82774264 | 0 | 0 |
| REACTOME_ACTIVATION_OF_THE_  PRE_REPLICATIVE_COMPLEX | | 30 | -0.8141718 | 0 | 0 |
| REACTOME_PROCESSIVE_SYNTHESIS_ON_THE_LAGGING_STRAND | | 15 | -0.80766445 | 0 | 2.89E-05 |
| REACTOME_ACTIVATION_OF_ATR_IN_RESPONSE_TO_REPLICATION_STRESS | | 35 | -0.80128485 | 0 | 0 |
| REACTOME_G2_M_CHECKPOINTS | | 41 | -0.7908015 | 0 | 0 |
| REACTOME_E2F_MEDIATED_  REGULATION_OF_DNA_REPLICATION | | 32 | -0.7828214 | 0 | 0 |
| REACTOME_KINESINS | | 20 | -0.7749984 | 0 | 0 |
| REACTOME_HOMOLOGOUS_  RECOMBINATION_REPAIR_OF_  REPLICATION_INDEPENDENT_DOUBLE_STRAND_BREAKS | | 16 | -0.7529953 | 0 | 7.11E-05 |
| REACTOME_G0_AND_EARLY_G1 | | 22 | -0.73598343 | 0 | 0 |
| REACTOME_PEPTIDE_CHAIN_  ELONGATION | | 83 | -0.7175654 | 0 | 0 |
| REACTOME_REGULATION_OF_  GLUCOKINASE_BY_GLUCOKINASE_  REGULATORY_PROTEIN | | 25 | -0.7153815 | 0 | 0 |
| REACTOME_NEP_NS2_INTERACTS_  WITH_THE_CELLULAR_EXPORT_  MACHINERY | | 27 | -0.71085745 | 0 | 0 |
| REACTOME_FORMATION_OF_THE_  TERNARY_COMPLEX_AND_  SUBSEQUENTLY_THE_43S_COMPLEX | | 48 | -0.70507 | 0 | 0 |
| REACTOME_TRANSPORT_OF_  RIBONUCLEOPROTEINS_INTO_THE  _HOST_NUCLEUS | | 27 | -0.7031889 | 0 | 0 |
| REACTOME_INFLUENZA_VIRAL_RNA_  TRANSCRIPTION_AND_REPLICATION | | 99 | -0.69562536 | 0 | 0 |
| REACTOME_METABOLISM_OF_NON  _CODING_RNA | | 48 | -0.69479805 | 0 | 0 |
| REACTOME_3_UTR_MEDIATED_  TRANSLATIONAL_REGULATION | | 103 | -0.6944623 | 0 | 0 |
| REACTOME_INTERACTIONS_OF_  VPR_WITH_HOST_CELLULAR_  PROTEINS | | 32 | -0.69361234 | 0 | 0 |
| REACTOME_MITOTIC_  PROMETAPHASE | | 84 | -0.6913364 | 0 | 0 |
| REACTOME_DNA_REPLICATION | | 184 | -0.6848482 | 0 | 0 |
| REACTOME_ACTIVATION_OF_THE_  MRNA_UPON_BINDING_OF_THE_CAP_BINDING_COMPLEX_AND_EIFS_AND_  SUBSEQUENT_BINDING_TO_43S | | 56 | -0.6823825 | 0 | 0 |
| REACTOME_INFLUENZA_LIFE_CYCLE | | 133 | -0.67958224 | 0 | 0 |
| REACTOME_MITOTIC_M_  M_G1_PHASES | | 165 | -0.6766168 | 0 | 0 |
| REACTOME_FANCONI_ANEMIA_  PATHWAY | | 21 | -0.67199504 | 0 | 4.55E-04 |
| REACTOME_FORMATION_OF_  TUBULIN_FOLDING_INTERMEDIATES_  BY_CCT_TRIC | | 20 | -0.6694275 | 0 | 2.81E-04 |
| REACTOME_NONSENSE_MEDIATED_  DECAY_ENHANCED_BY_THE_EXON_  JUNCTION_COMPLEX | | 103 | -0.66857463 | 0 | 0 |
| REACTOME_GLOBAL_GENOMIC_  NER_GG_NER | | 33 | -0.6680151 | 0 | 0 |
| REACTOME_PREFOLDIN_MEDIATED_  TRANSFER_OF_SUBSTRATE_TO_  CCT_TRIC | | 26 | -0.66648877 | 0 | 4.88E-05 |
| REACTOME_S_PHASE | | 104 | -0.6638708 | 0 | 0 |
| REACTOME_SYNTHESIS_OF_DNA | | 88 | -0.6585429 | 0 | 0 |
| REACTOME_SRP_DEPENDENT_  COTRANSLATIONAL_PROTEIN_  TARGETING_TO_MEMBRANE | | 106 | -0.656066 | 0 | 0 |
| REACTOME_G1_S_TRANSITION | | 105 | -0.65573084 | 0 | 0 |
| REACTOME_M_G1_TRANSITION | | 77 | -0.65304494 | 0 | 0 |
| REACTOME_TRANSPORT_OF_MATURE_MRNA_DERIVED_FROM_AN_  INTRONLESS_TRANSCRIPT | | 32 | -0.6520071 | 0 | 2.95E-05 |
| REACTOME_MRNA_SPLICING_  MINOR_PATHWAY | | 42 | -0.64506197 | 0 | 0 |
| REACTOME_RNA_POL_III_  TRANSCRIPTION_TERMINATION | | 19 | -0.6434694 | 0 | 0.001610112 |
| REACTOME_DOUBLE_STRAND_  BREAK_REPAIR | | 22 | -0.642346 | 0 | 0.001191476 |
| REACTOME_CELL_CYCLE_MITOTIC | | 304 | -0.6391397 | 0 | 0 |
| REACTOME_RNA_POL_I_  TRANSCRIPTION_TERMINATION | | 21 | -0.63748866 | 0 | 0.002091157 |
| REACTOME_TRANSLATION | | 142 | -0.6370052 | 0 | 0 |
| REACTOME_MITOTIC_G1_G1_S_  PHASES | | 129 | -0.63251984 | 0 | 0 |
| REACTOME_PROCESSING_OF_CAPPED_INTRONLESS_PRE_MRNA | | 23 | -0.6243813 | 0 | 0.001055595 |
| REACTOME_RNA_POL_III_CHAIN_  ELONGATION | | 17 | -0.623266 | 0.004514673 | 0.007013481 |
| REACTOME_G_BETA_GAMMA_  SIGNALLING_THROUGH_PLC_BETA | | 16 | -0.61838025 | 0 | 0.005880694 |
| REACTOME_APC_CDC20_MEDIATED_  DEGRADATION_OF_NEK2A | | 21 | -0.61837274 | 0 | 0.001675552 |
| REACTOME_ASSEMBLY_OF_  THE_PRE_REPLICATIVE_COMPLEX | | 62 | -0.6164791 | 0 | 0 |
| REACTOME_PHOSPHORYLATION_  OF_THE_APC_C | | 17 | -0.6159139 | 0.004640371 | 0.006144868 |
| REACTOME_GLUCOSE_TRANSPORT | | 32 | -0.61584157 | 0 | 1.31E-04 |
| REACTOME_CELL_CYCLE_  CHECKPOINTS | | 110 | -0.6068631 | 0 | 0 |
| REACTOME_CYTOSOLIC_TRNA_  AMINOACYLATION | | 24 | -0.6066835 | 0 | 0.001642803 |
| REACTOME_RESOLUTION_OF_AP_  SITES_VIA_THE_MULTIPLE_  NUCLEOTIDE_PATCH_  REPLACEMENT_PATHWAY | | 17 | -0.6065045 | 0.008752735 | 0.008734645 |
| REACTOME_INHIBITION_OF_THE_  PROTEOLYTIC_ACTIVITY_OF_APC_C_  REQUIRED_FOR_THE_ONSET_OF_  ANAPHASE_BY_MITOTIC_SPINDLE_  CHECKPOINT_COMPONENTS | | 18 | -0.6038739 | 0.00896861 | 0.008679578 |
| REACTOME_SMOOTH_MUSCLE_  CONTRACTION | | 22 | -0.60006946 | 0.002386635 | 0.004187366 |
| REACTOME_CDT1_ASSOCIATION_  WITH_THE_CDC6_ORC_ORIGIN_  COMPLEX | | 53 | -0.5979396 | 0 | 0 |
| REACTOME_TRANSPORT_OF_  MATURE_TRANSCRIPT_  TO_CYTOPLASM | | 52 | -0.59595996 | 0 | 2.84E-05 |
| REACTOME_PROCESSING_OF_CAPPED_INTRON_CONTAINING_PRE_MRNA | | 135 | -0.5951692 | 0 | 0 |
| REACTOME_MRNA_SPLICING | | 106 | -0.59391373 | 0 | 0 |
| REACTOME_MITOCHONDRIAL_  TRNA_AMINOACYLATION | | 21 | -0.5911081 | 0.005037783 | 0.00638738 |
| REACTOME_RNA_POL_III_  TRANSCRIPTION_INITIATION_  FROM_TYPE_3_PROMOTER | | 26 | -0.5903188 | 0.002439024 | 0.002414777 |
| REACTOME_TRNA_AMINOACYLATION | | 42 | -0.59024864 | 0 | 1.35E-04 |
| REACTOME_APC_C_CDC20_MEDIATED_DEGRADATION_OF_CYCLIN_B | | 19 | -0.59001476 | 0.002298851 | 0.008633464 |
| REACTOME_P53_INDEPENDENT_  G1_S_DNA_DAMAGE_CHECKPOINT | | 47 | -0.58805925 | 0 | 2.64E-05 |
| REACTOME_CDK_MEDIATED_  PHOSPHORYLATION_AND_  REMOVAL_OF_CDC6 | | 45 | -0.58700734 | 0 | 5.04E-05 |
| REACTOME_G_PROTEIN_BETA_  GAMMA_SIGNALLING | | 21 | -0.5868193 | 0.00486618 | 0.006103213 |
| REACTOME_ORC1_REMOVAL_FROM_  CHROMATIN | | 63 | -0.585339 | 0 | 0 |
| REACTOME_MITOTIC_G2_  G2_M_PHASES | | 77 | -0.5811044 | 0 | 0 |
| REACTOME_VIF_MEDIATED_  DEGRADATION_OF_APOBEC3G | | 48 | -0.5807526 | 0 | 4.96E-05 |
| REACTOME_MRNA_PROCESSING | | 153 | -0.58031523 | 0 | 0 |
| REACTOME_REGULATION_OF_  MITOTIC_CELL_CYCLE | | 75 | -0.57908165 | 0 | 0 |
| REACTOME_TRANSCRIPTION_  COUPLED_NER_TC_NER | | 44 | -0.5757114 | 0 | 1.37E-04 |
| REACTOME_FORMATION_OF_  THE_HIV1_EARLY_ELONGATION_COMPLEX | | 29 | -0.5725151 | 0 | 0.001688026 |
| REACTOME_RNA_POL_III_  TRANSCRIPTION | | 33 | -0.5704762 | 0 | 0.001169088 |
| REACTOME_REGULATION_OF_  ORNITHINE_DECARBOXYLASE_ODC | | 47 | -0.5663566 | 0 | 1.33E-04 |
| REACTOME_APC_C_CDC20_MEDIATED_  DEGRADATION_OF_  MITOTIC_PROTEINS | | 64 | -0.5619387 | 0 | 2.79E-05 |
| REACTOME_CYCLIN_E_ASSOCIATED_  EVENTS_DURING_G1_S_TRANSITION_ | | 61 | -0.5605064 | 0 | 2.74E-05 |
| REACTOME_METABOLISM_OF_RNA | | 250 | -0.55944085 | 0 | 0 |
| REACTOME_AUTODEGRADATION_  OF_THE_E3_UBIQUITIN_LIGASE_COP1 | | 46 | -0.55872524 | 0 | 3.16E-04 |
| REACTOME_APC_C_CDH1_MEDIATED_DEGRADATION_OF_CDC20_AND_OTHER_APC_C CDH1_TARGETED_PROTEINS_IN_LATE_  MITOSIS_EARLY_G1 | | 63 | -0.55845815 | 0 | 2.60E-05 |
| REACTOME_NUCLEOTIDE_  EXCISION_REPAIR | | 49 | -0.5555992 | 0 | 3.34E-04 |
| REACTOME_CLEAVAGE_OF_GROWING_TRANSCRIPT_IN_THE_TERMINATION_REGION_ | | 42 | -0.55287915 | 0 | 5.26E-04 |
| REACTOME_DNA_REPAIR | | 104 | -0.54827315 | 0 | 0 |
| REACTOME_SCFSKP2_MEDIATED_  DEGRADATION_OF_P27_P21 | | 52 | -0.54439205 | 0 | 2.42E-04 |
| REACTOME_MRNA_CAPPING | | 29 | -0.54394615 | 0.002358491 | 0.005664275 |
| REACTOME_RNA_POL_II_TRANSCRIPTION_  PRE_INITIATION_AND_PROMOTER_OPENING | | 40 | -0.53918284 | 0 | 0.001695035 |
| REACTOME_SCF_BETA_TRCP_  MEDIATED_DEGRADATION_OF_EMI1 | | 48 | -0.5389407 | 0 | 7.54E-04 |
| REACTOME_CELL_CYCLE | | 374 | -0.53856295 | 0 | 0 |
| REACTOME_HIV_LIFE_CYCLE | | 107 | -0.5332148 | 0 | 0 |
| REACTOME_G1_PHASE | | 35 | -0.5323318 | 0 | 0.004227321 |
| REACTOME_LOSS_OF_NLP_FROM_  MITOTIC_CENTROSOMES | | 56 | -0.52931577 | 0 | 4.00E-04 |
| REACTOME_FORMATION_OF_  RNA_POL_II_ELONGATION_COMPLEX_ | | 39 | -0.5285054 | 0 | 0.002454045 |
| REACTOME_METABOLISM_OF_MRNA | | 205 | -0.526802 | 0 | 0 |
| REACTOME_DESTABILIZATION_OF_  MRNA_BY_AUF1_HNRNP_D0 | | 49 | -0.522038 | 0 | 0.001689183 |
| REACTOME_AUTODEGRADATION_OF_  CDH1_BY_CDH1_APC_C | | 56 | -0.5180637 | 0 | 0.001030658 |
| REACTOME_HOST_INTERACTIONS_OF_  HIV_FACTORS | | 115 | -0.51601005 | 0 | 0 |
| REACTOME_CROSS_PRESENTATION_OF_  SOLUBLE_EXOGENOUS_  ANTIGENS_ENDOSOMES | | 42 | -0.5155201 | 0 | 0.003121394 |
| REACTOME_RECRUITMENT_OF_  MITOTIC_CENTROSOME_PROTEINS_AND_  COMPLEXES | | 63 | -0.51546013 | 0 | 3.68E-04 |
| REACTOME_LATE_PHASE_OF_  HIV_LIFE_CYCLE | | 97 | -0.5119963 | 0 | 2.56E-05 |
| REACTOME_MITOCHONDRIAL_  PROTEIN_IMPORT | | 48 | -0.5092736 | 0 | 0.003211276 |
| REACTOME_METABOLISM_OF_  NUCLEOTIDES | | 62 | -0.50759304 | 0 | 7.64E-04 |
| REACTOME_MUSCLE_CONTRACTION | | 34 | -0.50743794 | 0.002463054 | 0.007582443 |
| REACTOME_RNA_POL_II_  TRANSCRIPTION | | 97 | -0.50117356 | 0 | 1.17E-04 |
| REACTOME_RNA_POL_II_  PRE_TRANSCRIPTION_EVENTS | | 55 | -0.49347672 | 0 | 0.003166686 |
| REACTOME_HIV_INFECTION | | 182 | -0.49037874 | 0 | 0 |
| REACTOME_PROTEIN_FOLDING | | 50 | -0.48529267 | 0 | 0.004177411 |
| REACTOME_P53_DEPENDENT_  G1_DNA_DAMAGE_RESPONSE | | 52 | -0.46014234 | 0 | 0.007203301 |
| REACTOME_CHROMOSOME_  MAINTENANCE | | 97 | -0.4364906 | 0 | 0.003316784 |
| REACTOME_METABOLISM_OF_  PROTEINS | | 390 | -0.4287051 | 0 | 2.69E-05 |
| REACTOME_REGULATION_OF_MRNA_  STABILITY_BY_PROTEINS_THAT_  BIND_AU_RICH_ELEMENTS | | 80 | -0.42395088 | 0 | 0.008571514 |

**Repressive REACTOME pathways**

The table lists all the results of Reactome pathways analysis based on GSEA. Table A showed the activated REACTOME pathways in CAL27^AR^ cells. Table B showed the repressive REACTOME pathways in CAL27^AR^ cells.
